# A Novel Heptapeptide, GPPGPAG Transfers to the Brain, and Ameliorates Memory Dysfunction and Dendritic Atrophy in Alzheimer’s Disease Model Mice

**DOI:** 10.3389/fphar.2021.680652

**Published:** 2021-05-14

**Authors:** Chihiro Tohda, Chisato Kogure, Kaori Nomoto, Andreia de Toledo, Ximeng Yang, Eiichi Hirano

**Affiliations:** ^1^ Section of Neuromedical Science, Division of Bioscience, Institute of Natural Medicine, University of Toyama, Toyama, Japan; ^2^ Research Institute Japan Bio Products Co., Ltd., Kurume, Japan

**Keywords:** heptapeptide, BBB transfer, dendrite, alzheimer’s disease, memory recovery, 14-3-3ε, 5XFAD mouse

## Abstract

We investigated the effects of a heptapeptide, GPPGPAG, on memory improvement and neuritic regeneration in Alzheimer’s disease models to evaluate its potency as a new anti-Alzheimer’s disease (AD) therapy. The anti-AD effects of GPPGPAG were evaluated in Aβ-treated cortical neurons and 5XFAD, a mouse model of AD. Exposure of cortical neurons to Aβ25-35 for 3 days resulted in atrophy of axons and dendrites. Treatment with GPPGPAG improved the dendritic atrophy of Aβ-treated cortical neurons, but not axonal atrophy. Postsynaptic and presynaptic densities under Aβ1-42 exposure were increased by GPPGPAG post treatment. Oral administration of GPPGPAG to 5XFAD mice for 15 days improved significantly object recognition memory and dendritic density. Direct infusion of GPPGPAG into the lateral ventricle of 5XFAD mice for 28 days improved object recognition memory. Following oral administration of GPPGPAG in mice, the undigested heptapeptide was detected in the plasma and cerebral cortex. Analysis of target protein of GPPGPAG in neurons by DARTS method identified 14-3-3ε as a bound protein. The protective effect of GPPGPAG on Aβ1-42-induced dendritic atrophy was canceled by knockdown of 14-3-3ε. Taken together, these results suggest that GPPGPAG is orally available, transfers to the brain, and ameliorates memory dysfunction in AD brain, which is possibly mediated by 14-3-3ε-related dendritic restoration.

## Introduction

Alzheimer’s disease (AD) is still very challenging to treat; the current clinically prescribed medications treat the symptoms temporarily but cannot halt the progressive disorder. Since amyloid β (Aβ) oligomerization and/or deposition is the pathological cause of AD, lowering Aβ has been extensively studied as a strategy to inhibit AD. However, this strategy has not succeeded to date. In the brain of AD patients, Aβ deposition begins at an early stage, approximately 20–30 years before it is diagnosed. Aβ-driven neurite atrophy and synaptic loss begin at early stage and directly trigger memory deficit. Actually, synaptic loss is majorly observed more than neuronal death in autopsy samples of AD patients (Bertoni-Freddari et al., 2003).

We hypothesized that reconstructing the damaged neuronal network may help recover neuronal functions and focused on several short peptides which could inhibit Aβ-induced axonal and dendritic atrophy. Our previous study indicated that treatment with human placenta extract improved dendrite atrophy and memory impairment in AD model mice (Kogure and Tohda, 2017). Fractionation and exploring active constituents in the placenta extract suggested that fragments of collagen α1 chain could be rich ingredients because the extract was made by pepsin digestion (data not shown). We focused on a GPPGPAG sequence that frequently and repeatedly occurs in those fragments of collagen α1 chain. Although no other reports had investigated this heptapeptide GPPGPAG, we hypothesized that GPPGPAG could be a new neuroactive peptide. This study showed that the heptapeptide, GPPGPAG specifically extended dendrites after Aβ-induced atrophy. The biological activity and disease treating effect of this heptapeptide has not been reported earlier. Therefore, this study aimed to investigate the memory improvement effect of the new therapeutic peptide, GPPGPAG in 5XFAD, a mouse model of AD, its transport to the brain and molecular mechanism of action.

5XFAD mice used in this study overexpressed five familial AD mutations, acting together to additively increase levels of cerebral Aβ peptides, especially neurotoxic Aβ42 (Oakley et al., 2006). While the majority of AD transgenic mice take 6–12 months, or longer, to form amyloid plaques (Eriksen and Janus, 2007), 5XFAD mice begin to develop visible amyloid deposits as early as 2 months of age, which is consistent with their dramatically accelerated Aβ1-42 generation.

## Materials and Methods

All experiments were performed in accordance with the Guidelines for the Care and Use of Laboratory Animals of the Sugitani Campus of the University of Toyama. All protocols were carried out in compliance with the ARRIVE guidelines 2.0 and approved by the Committee for Animal Care and Use of the Sugitani Campus of the University of Toyama. The approval number for animal experiments is A2017INM-1 and the confirmation number for the gene recombination experiments is G2018INM-2. All efforts were made to minimize the number of animals used.

### Materials

GPPGPAG, GPPGPPG, GPPGPP, GPPGPA, PPGPAG, and GPP were synthesized by Japan Bio Services (Saitama, Japan). Purities of synthesized GPPGPAG, GPPGPPG, GPPGPP, GPPGPA, PPGPAG, and GPP were 95.18, 95.3, 97.46, 95.94, 95.4, and 95.94%, respectively. The active partial fragment of Aβ, Aβ25-35 (Sigma-Aldrich, St. Louis, MO, United States), was dissolved in sterile and distilled water and incubated for 4 days at 37°C. Full length Aβ1-42 was dissolved in dimethyl sulfoxide at 5 mM and diluted in Ham’s F-12 medium at 100 μM followed by incubation for 24 h at 4°C. The supernatant obtained after centrifuging for 10 min at 4°C was used as aggregated Aβ1-42.

### Animals

Transgenic mice (5XFAD) were obtained from The Jackson Laboratory (Bar Harbor, ME, United States). The 5XFAD mice have the following five mutations: Swedish (K670N and M671L), Florida (I716V), and London (V717I) in human APP695 cDNA and human PS1 cDNA (M146L and L286V) under the transcriptional control of the neuron-specific mouse Thy-1 promoter (Oakley et al., 2006). They were maintained by crossing hemizygous transgenic mice with B6/SJL F1 breeders. Wild-type mice were obtained by crossing a hemizygous 5XFAD mouse and a B6/SJL F1 mouse. To investigate the effect of GPPGPAG on 5XFAD, we used hemizygous 5XFAD mice (male or female, 5–7 months old) and non-transgenic littermate wild-type mice (male or female, 5–7 months old). ddY mice were purchased from Japan SLC (Shizuoka, Japan).

All mice were housed with free access to food and water and were maintained in a controlled environment (22 ± 2°C, 50 ± 5% humidity, 12 h light/dark cycle starting at 7:00 am).

### Primary Culture

Primary culture was performed as previously described (Tohda et al., 2012; Yang et al., 2017). Embryos were removed from ddY mice (Japan SLC, Shizuoka, Japan) at 14 days of gestation. The cortices were dissected and dura mater was removed. The tissues were minced, dissociated, and grown in cultures with neurobasal medium (Thermo Fisher Scientific, Waltham, MA, United States) that included 2% B-27 supplement (Invitrogen), 0.6% D-glucose, and 2 mML-glutamine in eight-well chamber slides (Corning, NY, United States) coated with 5 μg/ml poly-D-lysine at 37°C in a humidified incubator with 10% CO_2_. The seeding cell density was 2.2–2.9 × 10^4^ cells/cm^2^.

### Measurement of Dendritic and Axonal Lengths

**FIGURE 1:**
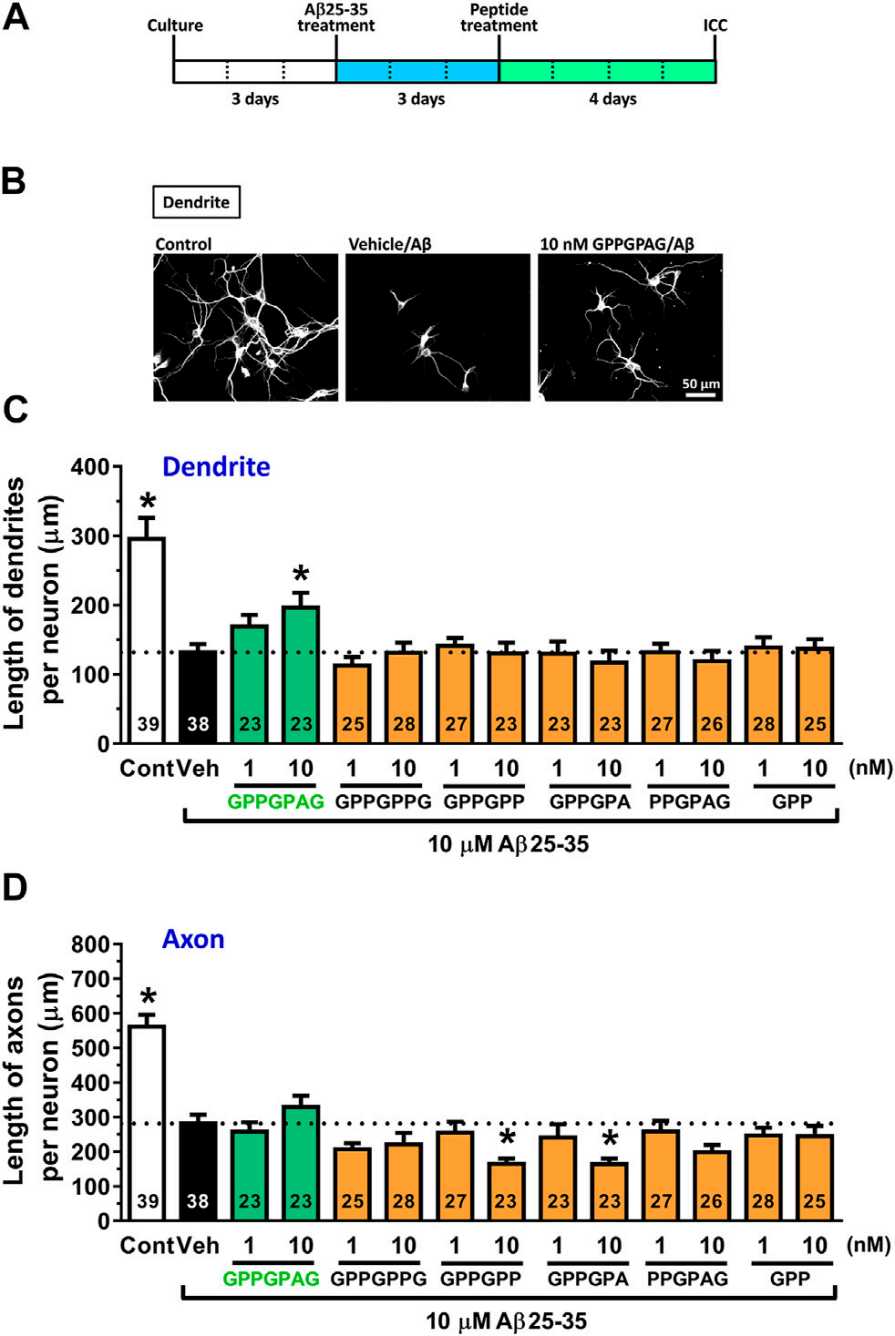
Effect of GPPGPAG on Aβ-induced atrophy of dendrites and axons **(A)** Cortical neurons were cultured for three days and then treated with or without aggregated Aβ25–35 (10 μM). Three days after the administration of Aβ25–35, the cells were treated with six types of peptide (GPPGPAG, GPPGPPG, GPPGPP, GPPGPA, PPGPAG, and GPP) at 1 and 10 nM concentration, or vehicle solution (Veh; distilled water). Four days after the treatment, the cells were fixed and double-immunostained for MAP2 and pNF-H. **(B)** Representative photos of the MAP2-positive dendrites are shown. The density of MAP2-positive dendrites **(C)** and pNF-H-positive axons **(D)** was quantified for each treatment. *p < 0.05, vs. Aβ25–35-treated vehicle solution group, one-way ANOVA with *post hoc* Bonferroni’s test, mean ± standard error. Each number of samples is shown in columns. Scale bar indicates 50 μm.

Evaluation of neurite length was performed as previously described (Tohda et al., 2012; Yang et al., 2017). For measurement of density of dendrites and axons, the cells were treated with or without 10 μM Aβ25-35 or 1 μM Aβ1-42 for 3 days and then treated with fresh medium containing GPPGPAG or the vehicle solution (distilled water) for 4 days. The neurons were fixed with 4% paraformaldehyde for 90 min and immunostained with a polyclonal antibody against microtubule-associated protein 2 (MAP2, 1:2,000, Abcam, Cambridge, United Kingdom) as a dendritic marker. A monoclonal antibody against phosphorylated neurofilament-H (pNF-H) (1:250, SMI-35, BioLegend, San Diego, CA, United States) was used as an axonal marker. Alexa Fluor 594-conjugated goat anti-rabbit IgG (1:600) and Alexa Fluor 488-conjugated goat anti-mouse IgG (1:600) were used as the secondary antibodies (Molecular Probes, Eugene, OR, United States). Nuclear counterstaining was performed using DAPI (1 μg/ml, Sigma-Aldrich). The fluorescence images were captured with a 10X objective lens using a fluorescence microscope system (Cell Observer, Carl Zeiss, Tokyo, Japan). Twenty-three to thirty-nine images (Figure 1) were captured per treatment. The lengths of the MAP2-positive dendrites and pNF-H-positive axons were measured using a MetaMorph analyzer (Molecular Devices, Sunnyvale, CA, United States), which automatically traces and measures the neurite length without measuring the cell bodies. The sum of the dendrite or axon length was divided by the number of MAP2-positive neurons.

### Presynaptic and Postsynaptic Density

**FIGURE 2:**
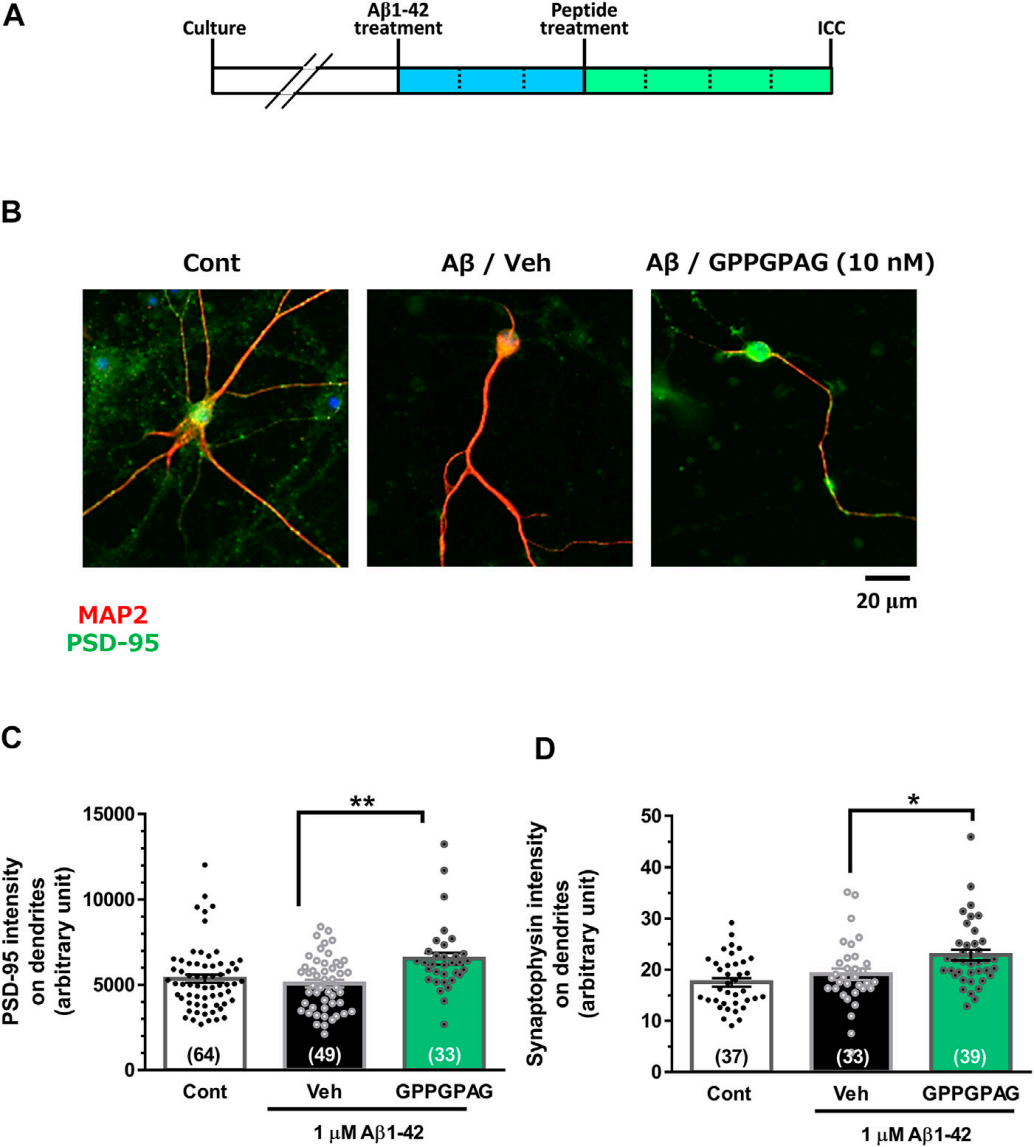
Effect of GPPGPAG on post-synaptic and pre-synaptic intensities under Aβ treatment. **(A)** Cortical neurons were cultured for 21 days and then treated with or without aggregated Aβ1-42 (1 μM). Three days after the administration of Aβ1-42, the cells were treated with GPPGPAG or a vehicle solution (Veh; distilled water). After an additional 4-days of treatment, the cells were fixed and double-immunostained for MAP2 and PSD-95 or MAP2 and synaptophysin. **(B)** Representative photos of the PSD-95-positive presynaptic vesicles (green) on MAP2-positive dendritic shafts (red) are shown. Scale bar indicates 20 μm. **(C)** The PSD-95-positive puncta intensity was quantified for each dendrite. **p < 0.01, one-way ANOVA with *post hoc* Bonferroni’s test, *n* = 33–64 **(D)** The synaptophysin-positive puncta intensity was quantified for each dendrite. *p < 0.05, one-way ANOVA with *post hoc* Bonferroni’s test, *n* = 33–39. mean ± standard error. Each number of samples is shown in columns.

Neural cells were seeded at 2.2 × 10^4^ cells/cm^2^ (Figure 2) or 1.5 × 10^4^ cells/cm^2^ (Supplementary Figure S1). After 21 days of culture, the medium was replaced with a serum-free medium containing 1 μM Aβ1-42 (Figure 2) or 10 μM Aβ25-35 (Supplementary Figure S1). After 3 days, the entire medium was replaced with a serum-free medium containing a drug or solvent. After another 4 days, the cells were fixed and 100 μL of the primary antibody solution [0.3% Triton X-100-PBS solution, normal goat serum, rabbit anti-PSD-95 monoclonal antibody (1:200, D27E11, Cell Signaling)] or [0.3% Triton X-100-PBS solution, normal goat serum, mouse anti-synaptophysin monoclonal antibody (1: 500, SVP-38, Sigma-Aldrich), and mouse anti-MAP2a and 2b monoclonal antibody (1:300, AP20, Merck, Darmstadt, Germany) or rabbit anti-MAP2 polyclonal antibody (1: 2,000, Abcam)] was added, followed by incubation at 4°C, overnight. The next day, the solution was removed and 100 μL of the secondary antibody solution [0.3% Triton X-100-PBS solution, Alexa Fluor 594-labeled goat anti-mouse IgG PLUS antibody (1: 1,000, Molecular Probes) and Alexa Fluor 488-labeled goat anti-rabbit IgG PLUS (1: 1,000, Molecular Probes)] or [0.3% Triton X-100-PBS solution, Alexa Fluor 488-labeled goat anti-mouse IgG PLUS antibody (1: 1,000, Molecular Probes) and Alexa Fluor 594-labeled goat anti-rabbit IgG PLUS (1: 1,000, Molecular Probes)] was added, followed by incubation at room temperature for 2 hours in the dark. Simultaneously, nuclear counterstaining was performed using DAPI (1 μg/ml). After washing with PBS for 5 min, it was sealed with Aqua Poly Mount. Fluorescence images were taken using BZ-X700 microscope (Keyence, Osaka, Japan) or Cell Observer microscope (Carl Zeiss). Images were obtained with a 20x or a 40x objective lens. The length of the MAP2-positive dendrites were measured by MetaMorph analyzer (Molecular Devices). The intensity of PSD-95-positive or synaptophysin-positive puncta merged on the dendrites (MAP2-positive neurites) were automatically traced and quantified using MetaMorph analyzer (Molecular Devices). The postsynaptic or presynaptic intensity per unit length of dendrites was shown.

### Administration of GPPGPAG

For intraventricular administration, GPPGPAG was dissolved in artificial cerebrospinal fluid (ACSF; 130 mM NaCl, 24 mM NaHCO_3_, 3.5 mM KCl, 1.3 mM Na_2_HPO_4_, 2 mM CaCl_2_, 2 mM MgCl_2_ 6H_2_O, and 10 mM glucose at pH 7.4) for use. GPPGPAG or ACSF was continuously injected for 28 days into the left ventricle (bregma: −0.2 mm, lateral: 1.0 mm, depth: −3.0 mm) using Alzet Osmotic Pump (1004, DURECT Corporation, CA, United States) and ALZET Brain Infusion Kit 3 (DURECT Corporation). Based on the amount of CSF produced which was 18 μL/h and the pump flow rate of 0.11 μL/h, the peptide in the pump was estimated to be diluted 164 times in the CSF to reach a final concentration 10 nM which was the effective dose in the cultured neuron experiments. Therefore 1.64 µM GPPGPAG was loaded into the pump. Stability of GPPGPAG in saline, mouse plasma and mouse cerebral cortex was confirmed by LC-MS quantification. GPPGPAG was mixed with saline or plasma at 5 μg/ml concentration. Otherwise, GPPGPAG was mixed with cortical lysate at 6.5 μg/g of cortex. After incubation at 37°C for 0, 10, 60, and 180 min, samples were dried up and served to LC-MS. Areas of MS peaks of GPPGPAG were quantified.

For oral administration; GPPGPAG (1, 10 mg/kg/day) or a solvent (saline) was orally administered once a day, totally for 26 days.

### Object Recognition Memory Test

**FIGURE 3:**
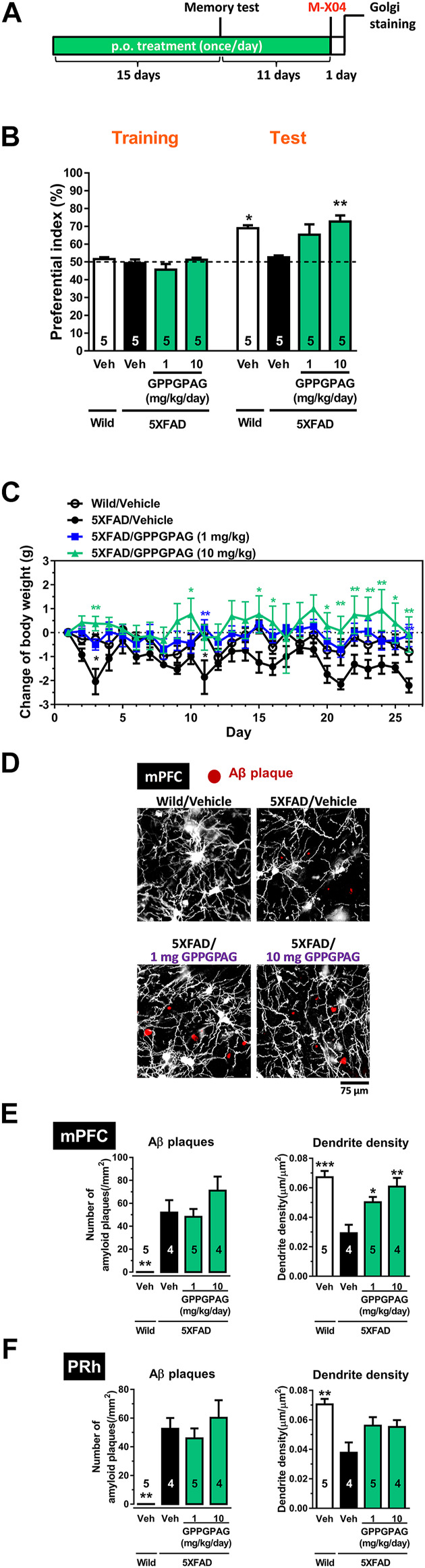
Effect of oral administration of GPPGPAG on object recognition memory deficit and dendritic atrophy in 5XFAD mice **(A)** GPPGPAG (1 and 10 mg/kg/day, p. o.) or a vehicle solution (physiological saline) was administered for 15 days to mice (male, 5–6 months old). Sixty minutes after the last administration, an object recognition test was carried out with a 3 h interval time between a training session and test session. **(B)** The preferential indices of the training and test sessions are shown. *p < 0.05, **p < 0.01 vs. vehicle-treated 5XFAD mice, repeated measures two-way ANOVA with *post hoc* Bonferroni’s test, mean ± standard error, *n* = 5 mice. **(C)** Changed values of body weights of mice during the experimental period is shown. *p < 0.05, **p < 0.01 vs. vehicle-treated 5XFAD mice, repeated measures two-way ANOVA with *post hoc* Bonferroni’s test. mean ± standard error, *n* = 5 mice. **(D)** Additional 11 days treatment was continued, then whole brains were Golgi stained to visualize the dendrites. Twenty-four hours before the removal of the brain, methoxy-X04 was i. p. injected to visualize brain Aβ deposition. Representative photos of medial prefrontal cortex (mPFC) slices are shown. Red spots are methoxy-X04-positive Aβ plaques. Aβ plaques number and dendrite density around the Aβ plaques were quantified in the mPFC **(E)** and perirhinal cortex (PRh) **(F)**. *p < 0.05, **p < 0.01, ***p < 0.001 vs. vehicle-treated 5XFAD mice, one-way ANOVA with *post hoc* Bonferroni’s test, *n* = 4–5 mice.

**FIGURE 4:**
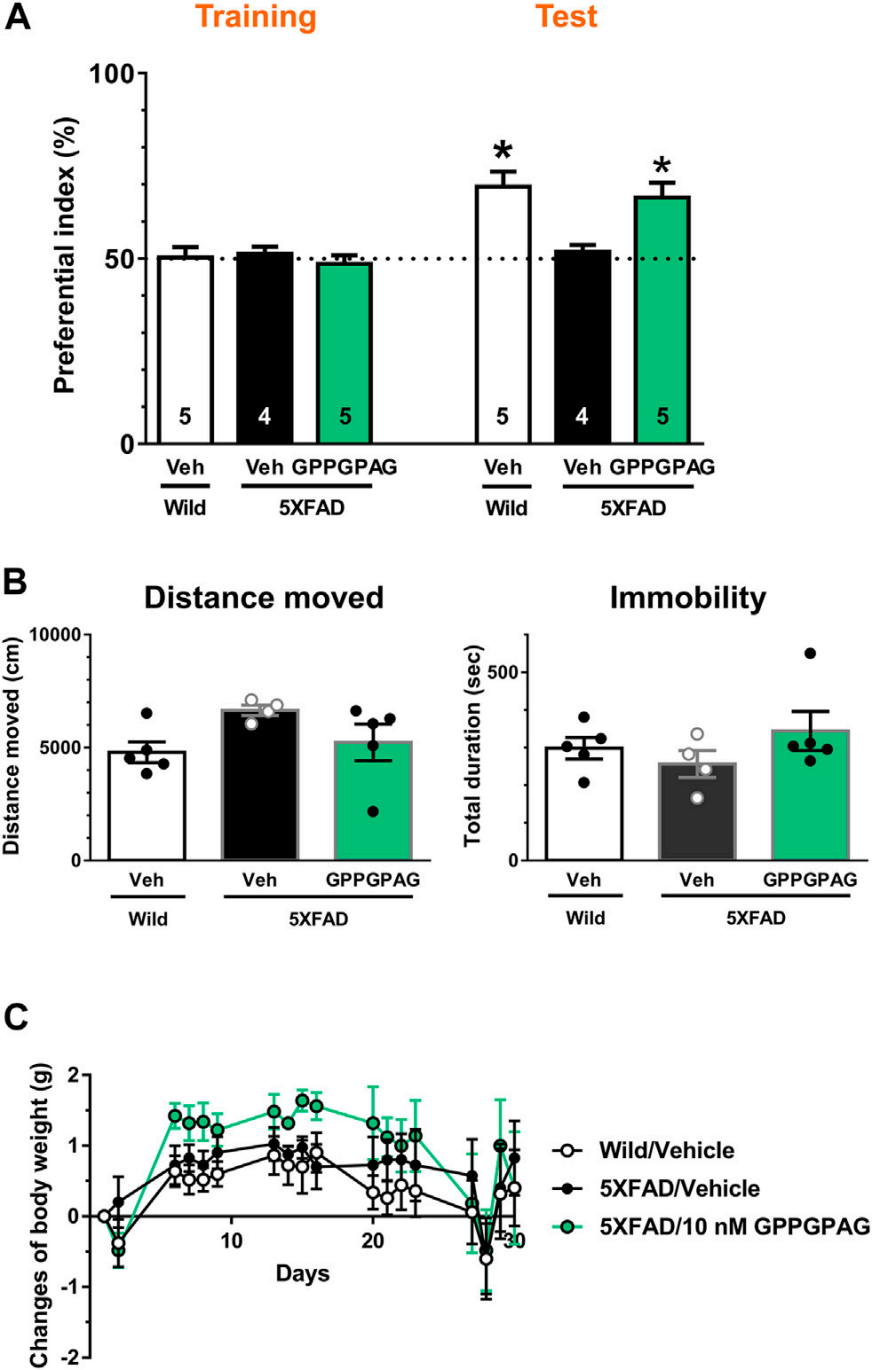
Effect of i.c.v. infusion of GPPGPAG on object recognition memory deficits in 5XFAD mice **(A)** GPPGPAG or vehicle solution (artificial cerebrospinal fluid; ACSF) was continuously administered to the lateral ventricle for 28 days in mice (male and female, 24–27 weeks old). The dose of GPPGPAG was set as 10 nM in CSF. An object recognition test was carried out with a 3 h interval time. The preferential indices of the training and test sessions are shown. *p < 0.05 vs. vehicle-treated 5XFAD mice, repeated measures two-way ANOVA with *post hoc* Bonferroni’s test. mean ± standard error. Wild-type (male, *n* = 5), vehicle-treated 5XFAD mice (male, *n* = 2; female, *n* = 2), GPPGPAG-infused 5XFAD mice (male, *n* = 2; female, *n* = 3). **(B)** Locomotion was evaluated by open field test. Distance moved and duration with immobility were shown. mean ± standard error **(C)** Changed values of body weight of mice during whole experimental period are shown. Repeated measures two-way ANOVA revealed no significant difference among four groups. mean ± standard error.

The object recognition memory test was performed as previously described (Tohda et al., 2012; Yang et al., 2017). The test was executed 15 days after oral administration (Figure 3) or 28 days after intraventricular administration (Figure 4; Supplementary Figure S2) of GPPGPAG. The mice were individually habituated to a polyvinyl chloride open-field box (49 cm × 49 cm; height, 48.5 cm) for 10 min. A novel object recognition test was performed. Two identical objects (colored ceramic ornaments) were placed at a fixed distance within a square box (30 cm × 40 cm; height, 36.5 cm, 66–97 lux). A mouse was then placed at the center of the box, and the number of times it made contact with the two objects was recorded during a 10 min period (training session). The mice were then placed back into the same box 3 h after the training session, and one of the objects used during the training session was replaced with a novel object (another ceramic ornament with a different shape and color). The mice were then allowed to explore freely for 10 min; the number of times they made contact with each object was recorded (test session). A preference index was used to measure cognitive function for objects. The index was defined as the ratio of the number of times a mouse made contact with one of the objects (training session) or the replaced novel object (test session) over the total number of times the mouse made contact with both objects.

### Locomotion Test

The day after the object recognition memory test, each mouse was gently released in the center of the black box and allowed to move freely for 10 min. Block color open box (49 cm × 49 cm; height, 48.5 cm, 66–97 lux) was used for the locomotion test. The locus of the mouse in the box was recorded with a digital camera. The total travel distance for 10 min was analyzed with EthoVision 3.0 (Noldus, Wageningen, Netherlands).

### Methoxy-X04 Staining

To visualize the amyloid plaques, methoxy-X04 (10 μg/kg, Abcam) was intraperitoneally administered once on the last day of oral administration, and the whole brain was removed after 24 h and stained by Golgi Method (Golgi Staining). Z-stack images of bright-field images and Aβ-stained images were taken with a 20x objective lens (1.0 µm pitch, 20 images) using the fluorescence inverted microscope (BZ-X700, Keyence) and subjected to full focus processing.

### Golgi Staining

Golgi staining was performed as previously described (Kogure and Tohda, 2017). Mice were anesthetized by intraperitoneal administration of a mix of three anesthetics (0.75 mg/kg of medetomidine (ZENOAQ, Fukushima, Japan), 4.0 mg/kg of midazolam (Sandoz, Tokyo Japan), and 5.0 mg/kg of butorphanol (Meiji Seika Pharma, Tokyo, Japan). The whole brain was removed from the skull without perfusion, and Golgi staining was performed according to the protocol using the FD Rapid GolgiStain Kit (FD NeuroTechnologies, Columbia, MD, United States). After soaking in 50% solution A-50% solution B for 2 weeks, the brain was replaced with Solution C, soaked for 3–7 days, and rapidly frozen with dry ice. Serial coronal slices of 200 μm-thickness of the prefrontal cortex (bregma +1.70 to +2.46 mm) and parietal cortex (bregma −1.34 to −2.06 mm) were prepared using a cryostat (CM3050S; Leica Microsystems, Tokyo, Japan). The slices were washed twice with sterilized purified water and then stained with 25% solution D-25% solution E aqueous solution for 15 min. The slides were dehydrated with a graduated ethanol series and xylene and then coverslipped with mounting medium (Mount-Quick, Daido Sangyo, Tokyo, Japan). Bright field images for dendrites were captured with a 20X objective lens using an all-in-one microscope (BZ-X700, Keyence). In the dendrite-stained photos, the areas associated with Aβ plaques were encircled as Regions of Interest (ROI) with a diameter of 75 µm centered on Aβ (Methoxy-X04-positive) plaque. The dendrite lengths in the ROI were quantified using Neurocyte software (Toyobo, Osaka, Japan), which automatically traces and measures neurite length without measuring the cell bodies. Total length inside the cropped circle (75-µm diameter) was defined as the dendrite density per unit area.

### Bioavailability and Brain Penetration of GPPGPAG

Bioavailability and brain penetration of GPPGPAG was performed as previously described (Yang et al., 2017). GPPGPAG (500 mg) was dissolved in 750 μL of physiological saline. GPPGPAG or vehicle solution (physiological saline) was orally administered using an oral gavage to female ddY mice (8 weeks-old). The mice were euthanized at 10, 60, and 180 min after administration. Blood was collected and centrifuged at 10,000 g and 4°C for 10 min to yield plasma. Plasma aliquots (200 μL) were extracted with methanol, dried, sonicated, and resolubilized in 50% methanol (100 μL). The cerebral cortex was also collected after complete saline perfusion for removing blood. The cortex was homogenized, extracted with methanol, dried, sonicated, and resolubilized in 50% methanol (100 µL). To calculate GPPGPAG concentration in the plasma and brain using liquid chromatography-mass spectrometry (LC-MS), a standard curve was generated by analyzing standard amounts of GPPGPAG solubilized in methanol at a dosage of 1, 5, and 10 μg/ml. A Thermo Scientific Accela high-performance LC (HPLC) system, interfaced with an LTQ Orbitrap XL hybrid Fourier Transform Mass Spectrometer (Thermo Fisher Scientific), was used to chemically profile GPPGPAG and the biosamples. The LC analysis was performed on a InertSustain AQ-C18 (4.6 mm ID × 50 mm, 5 μm, GL Sciences, Tokyo, Japan) held at 40°C with a flow rate of 200 μL/min 0.01% formic acid in water (a) and methanol (b) were used in the mobile phase, with the following linear elution gradient: 0–15 min, 10–90% b; 15–18 min, 90–10% b; 18–20 min, 10% b. The following electrospray interface (ESI) parameters were used: spray voltage, 4.5 kV; capillary voltage, 40.0 kV; tube lens, 150 V; capillary temperature, 330°C; sheath gas flow rate, 50 units; aux gas flow rate, 10 units. We operated the mass spectrometer in the positive ESI mode with scanning from 50 to 2,000 m/z and calibrated the instrument using a polytyrosine solution before each experiment.

### Identification of Putative GPPGPAG Direct Binding Protein in Cultured Neurons by Drug Affinity Responsive Target Stability Analysis

**FIGURE 5:**
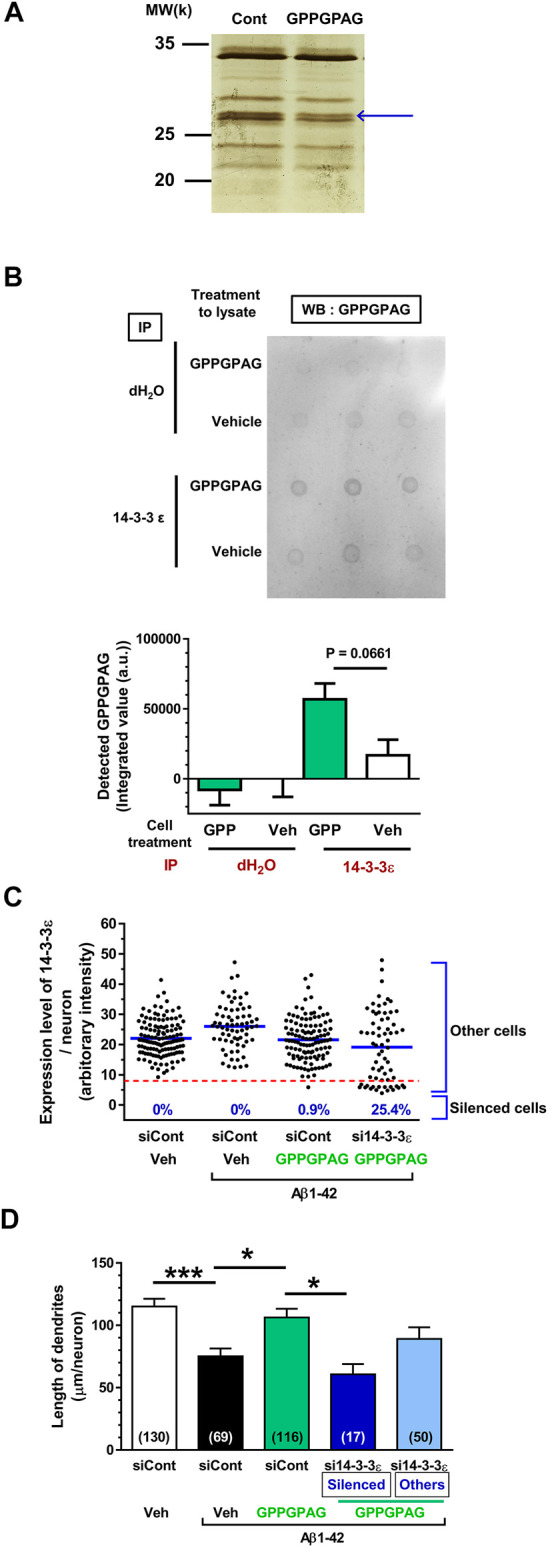
Direct binding protein of GPPGPAG in the neurons is 14-3-3ε. **(A)** Mouse cortical neuron primary cultures (ddY, E14) were treated by vehicle solution or GPPGPAG (10 nM) for 30 min. Cell lysate was used in DARTS reaction and the separated proteins were silver stained. **(A)** A band in the GPPGPAG-treated neuron that was thinner than in the vehicle solution-treated neurons (Cont), was cut out and analyzed by nanoLC-MS/MS (blue arrow). Full gel image is shown in Supplementary Figure S4. **(B)** To confirm direct binding of GPPGPAG with 14-3-3ε, vehicle solution or GPPGPAG (10 nM)-treated neuron lysate was immunoprecipitated with anti-14-3-3ε antibody, and then dot blotted by anti-GPPGPAG antibody. Quantified data are shown. Unpaired *t*-test was performed, mean ± standard error, *n* = 3. **(C)** Knockdown of 14-3-3ε by siRNA transfection in cortical neurons. 2 days after the culture of cortical neurons, siRNA of 14-3-3ε or control siRNA was transfected using Magnetofection. Drug treatment was started the next day. Treatment with or without aggregated Aβ1-42 at 1 μM, and with or without 10 nM GPPGPAG was continued for 4 days. Expression level of 14-3-3ε was quantified in each neuron and plotted. An intensity level of 14-3-3ε under eight was classified as silenced cells, and intensity higher than eight was classified as other cells. **(D)** The length of MAP2-positive dendrites was quantified for each treatment. *p < 0.05, ***p < 0.001, one-way ANOVA with *post hoc* Bonferroni’s multiple comparisons test, mean ± standard error, Each number of samples is shown in columns.

DARTS analysis was performed as previously described (Tohda et al., 2012). Cortical neurons was cultured for 3 days, the cells were treated by vehicle solution-contained medium or 10 nM GPPGPAG-containing medium for 30 min at 37°C. Cells were washed with PBS and then incubated with mammalian protein extraction reagent (M-PER) lysis buffer (Thermo Fisher Scientific) containing a protease and phosphate inhibitor cocktail (Thermo Fisher Scientific) for 20 min on ice. After incubation, the cell solution was centrifuged (14,000 × g, 10 min, 4°C) to remove the cell debris, and the supernatants were used as drug-treated cell lysates. The cell lysate protein concentration was measured using a Pierce 660 nm Protein Assay Kit (Thermo Fisher Scientific). Cell lysate of cultured cortical neurons containing 3.1 μg protein was proteolysed with 0.5 μg thermolysin (Wako) in a reaction buffer containing 50 mM Tris-HCl, pH 8.0; 50 mM NaCl; 10 mM CaCl_2_ for 30 min at 37°C. At the end of the reaction period, 0.5 M ethylenediaminetetraacetic acid (pH 8.0) was added to each sample at a 1:10 ratio on ice to stop the proteolysis. Samples were incubated with NuPAGE LDS sample buffer (Life Technologies, Carlsbad, CA, United States) and 5% 2-mercaptoethanol at 75°C for 5 min. The samples were loaded onto 10% gradient polyacrylamide gels and electrophoresed. The gels were incubated in fixative solution (40% ethanol, 10% acetic acid in ultrapure water) at room temperature overnight. The proteins in the gels were silver stained for visualisation using a SilverQuest Kit (Thermo Fisher Scientific). One protein band (indicated by the blue arrows in Figure 5A) was thinner in the sample treated with GPPGPAG compared to that of the sample treated with vehicle solution. The band was excised from the gel, digested with trypsin, and then analyzed by mass spectrometry using a Nano liquid chromatography-tandem mass spectrometry (nanoLC-MS/MS) system (Japan Bio Services, Saitama, Japan). The candidate protein from the electrophoresis band was identified as 14-3-3ε using MASCOT database and the spectrum data. To confirm whether the candidate protein was 14-3-3ε, immunoprecipitation and dot blotting, or western blotting was performed after the DARTS reaction.

### Immunoprecipitation and Dot Blotting

Immunoprecipitation was performed as previously described (Yang et al., 2017). Cortical neurons were cultured for 3 days, the cells were treated by medium containing vehicle solution or 10 nM GPPGPAG for 60 min at 37°C. Cell lysate was prepared as described earlier. Cell lysate (30 μg) and rabbit antibody for 14-3-3ε (1:4,000, ProteinTech, Rosemont, IL, United States) were co-incubated for 1 h at 4°C with rotation. 30 mL of Dynabeads Protein G (Life technologies) was treated with 1% bovine serum albumin (BSA) in PBS for blocking for 2 h at room temperature with rotation. Then protein G was incubated with the lysate plus antibody solution for 10 min at 4°C with rotation. After washing protein G by 0.02% Tween 20 in tris buffered saline (T-TBS), each sample was eluted by NuPAGE LDS sample buffer and was heated for 5 min at 65°C. The samples were spotted onto a nitrocellulose membrane (Bio-Rad, Berkeley, CA, United States). After dried up, the membrane was blocked by 5% nonfat dry milk (Wako, Tokyo, Japan). Subsequently, the membrane was briefly washed with 0.1% T-TBS and incubated with the primary rabbit antibody against GPPGPAG (1:500, Scrum, Tokyo, Japan) in immunoreaction buffer (Can Get Signal solution 1) overnight at 4°C. After washing with 0.1% T-TBS, the membrane was incubated with a horse radish peroxidase (HRP)-conjugated secondary antibody (goat anti-rabbit IgG-HRP, Santa Cruz) at 1:2,000 dilution in immunoreaction buffer (Can Get Signal solution 2) for 1 h at room temperature with rotation. Dots were visualised with Amersham ECL detection regent (GE Healthcare) and detected by LAS4000 (GE Healthcare). Intensities of dots were quantified using CS analyzer (ATTO, Tokyo, Japan).

### Western Blotting

Western blotting was performed as previously described (Yang et al., 2017). Samples after proteolysis in the DARTS analysis were served for Western blotting. Samples after the DARTS reaction were mixed with NuPAGE lithium dodecyl sulphate (LDS) sample buffer (Life Technologies) containing 5% 2-mercaptoethanol (Wako, Osaka, Japan) at 75°C for 5 min and loaded onto an 14% sodium dodecyl sulphate-polyacrylamide gel (SDS–PAGE). After electrophoresis, proteins in the gel were transferred to a nitrocellulose membrane (Bio-Rad) and blocked with 0.1% T-TBS containing 5% skim milk (Wako) at room temperature. Subsequently, the membrane was gently washed with T-TBS and incubated with a mouse monoclonal anti-14-3-3β/ε/ζ antibody (1:1,000, clone 3C8, Abcam) in Can Get Signal solution 1 (Toyobo) overnight at 4°C. After washing with 0.1% T-TBS, the membrane was incubated with a Horse-Radish Peroxidase (HRP)-conjugated secondary antibody against mouse IgG (1:2,000; Cat. No. sc-2005, Santa Cruz) in Can Get Signal solution 2 (Toyobo) for 2 h at room temperature. After washing, the membrane was allowed to react with ECL Prime Western Blotting Detection Reagent (GE Healthcare) and chemiluminescence on the membrane was detected using an ImageQuant LAS 4000 system (GE Healthcare). Intensities of bands were quantified using CS analyzer (ATTO, Tokyo, Japan).

### siRNA Transfection

As siRNA of 14-3-3ε, Mission esiRNA ywhae (Sigma-Aldrich, Cat. No. EMU089421) was purchased. As control siRNA, Mission esiRNA RLUC (Sigma-Aldrich, Cat. No. EHURLUC) was purchased. Transfection was performed using Magnetofection (OZ Biosciences, San Diego, CA, United States) according to manufacturer’s protocol. siRNA was transfected 2 days after the culture, and drug treatment was started on the next day. Cells were treated with aggregated Aβ1-42 at 1 μM, and 10 nM GPPGPAG for 4 days. The concentration of siRNA was set at 200 nM based on preliminary examination. For quantification of 14-3-3ε expression level, immunocytochemistry was performed using a rabbit polyclonal anti-14-3-3ε antibody (1:100, Cat. No. 11648-2-AP, Proteintech, Rosemont, IL, United States), and a Alexa Fluor 594-conjugated goat anti-rabbit IgG (1:1,000) secondary antibody. Nuclear counterstaining was performed using DAPI (1 μg/ml, Sigma-Aldrich). Dendrites were immunostained using a mouse monoclonal MAP2a, 2 b (1:300, Thermo Fisher Scientific), and Alexa Fluor 488-conjugated goat anti-mouse IgG secondary antibody (1:1,000).

### Statistical Analysis

Data was expressed as mean ± standard error. To determine the statistically significant differences, GraphPad Prism 6 (GraphPad Software, Sun Diego, CA, United States) was used for statistical analyses. Used tests are One-way analysis of variance (ANOVA), *post hoc* Bonferroni's multiple comparisons test, repeated measures two-way ANOVA, *post hoc* Bonferroni's multiple comparisons test, and two-tailed unpaired *t*-test. The significance level was set at 5%.

## Results

### GPPGPAG Restores Aβ-Induced Dendritic Atrophy

The Aβ-induced dendritic and axonal atrophy models were used to evaluate the neurite-repairing activity of test drugs. Because our previous studies indicated effects in this model corelated well to memory improvement effects *in vivo*. The test drugs that showed beneficial effects in the cellular assays were also effective for memory recovery in AD model mice (Joyashiki et al., 2011; Tohda et al., 2012; Yang et al., 2017).

Three days after Aβ25–35 treatment, each peptide was added to cortical neurons at 1 and 10 nM. After 4 days of treatment, the dendrites and axons were quantified by immunostaining for MAP2 (dendritic marker) and pNF-H (axonal marker) (Figure 1A). Aβ25–35 administration (black columns) significantly decreased lengths of dendrites and axons compared with Aβ25–35-free treatment (white columns). However, GPPGPAG treatment (green columns) dose-dependently and significantly increased the dendritic length (Figures 1B,C) but not axonal density (Figure 1D). Other similar sequences GPPGPPG, GPPGPP, GPPGPA, PPGPAG, and GPP showed no effect on dendrite and axonal regrowth (Figures 1C,D).

To assess repairing synaptic loss, we quantified the expression intensity of PSD-95-positive postsynaptic puncta or synaptophysin-positive presynaptic puncta in long-term cultured cortical neurons under Aβ1-42 treatment (Figure 2A). Postsynaptic density (Figures 2B,C) and presynaptic density (Figure 2D) were significantly increased by GPPGPAG (10 nM). Synaptophysin-positive presynaptic areas were significantly decreased by Aβ25–35, and GPPGPAG (1, 10, and 100 nM) significantly increased the synaptic areas (Supplementary Figure S1).

### GPPGPAG Ameliorates Memory Impairment and Dendritic Loss in AD Model Mice

To investigate the effect of GPPGPAG on impaired object recognition memory in 5XFAD mice (male, 25–28 weeks old), we administered GPPGPAG (1 and 10 mg/kg/day) or vehicle solution orally to mice for 15 days. After 15 days of administration, an object recognition test was performed (Figure 3A). We determined that 3 h was the appropriate time of interval between a training session and a test session in which 5XFAD mice forgot object information. In the training session, all four groups showed approximately 50% preferential index. In the test session, wild-type mice and GPPGPAG (1 and 10 mg/kg/day)-treated 5XFAD mice showed significantly high exploratory behavior against a novel object (Figure 3B). In contrast, the exploratory behavior toward the novel object in the vehicle-treated 5XFAD mice was close to chance (50%). Repeated measures two-way ANOVA indicated significant drug × test interaction as follows.

• vehicle solution-treated wild-type vs. vehicle solution-treated 5XFAD [F (1, 8) = 19.72, P = 0.022]
• 1 mg/kg GPPGPAG-treated 5XFAD vs. vehicle solution-treated 5XFAD [F (1, 8) = 15.10, P = 0.046]
• 10 mg/kg GPPGPAG-treated 5XFAD vs. vehicle solution-treated 5XFAD [F (1, 8) = 24.10, P = 0.0012].

*Post hoc* Bonferroni’s multiple comparisons test also indicated significant improvement of object recognition memory in 1 mg/kg and 10 mg/kg GPPGPAG as well as wild-type mice (Figure 3B). Body weights of vehicle solution-treated 5XFAD mice was slightly reduced compared with wild-type mice [drug × test interaction; F (25,200) = 1.841, *p* = 0.0116]. 1 mg/kg GPPGPAG administration normalized body weights [drug × test interaction; F (25,200) = 2.067, *p* = 0.0032] (Figure 3C). 10 mg/kg GPPGPAG administration also normalized body weights [drug × test interaction; F (25,200) = 1.873, *p* = 0.0097] (Figure 3C). When compared with vehicle solution-treated 5XFAD mice, *post hoc* Bonferroni’s multiple comparisons test indicated significant increases in body weight at day 3 and 11 in vehicle solution-treated wild-type mice, at day 11 and 26 in 1 mg/kg GPPGPAG group, and at day 3, 9, 15, 16, 20–26 in 10 mg/kg GPPGPAG group (Figure 3C).

Administration of the GPPGPAG or vehicle solution was continued further for 11-days after the memory test, the mouse brains were removed, and the dendrites were specifically visualized by Golgi staining. Since the prefrontal cortex and perirhinal cortex are mainly related to object recognition memory (Warburton and Brown, 2015) the dendrite density in both areas were evaluated. Twenty-four hours before sacrificing, methoxy-X04 was i. p. injected to visualize the Aβ plaques *in vivo*. In the Golgi stained brain slices, dendrite length within a concentric circle of radius 75 μm from the methoxy-X04-positive Aβ plaques were quantified. Dendrite density in the vehicle-treated 5XFAD mice was decreased compared to that in wild-type mice (Figures 3D–F). In contrast, GPPGPAG administration significantly increased the dendritic density in the prefrontal cortex. The number of Aβ plaques were not changed by GPPGPAG administration in both areas (Figures 3E,F). In the hippocampus, the proper evaluation was not impossible because densely overlapped staining of dendrites. Staining of one mouse in vehicle solution-treated 5XFAD mice and one mouse in 10 mg/kg GPPGPAG-treated 5XFAD mice was technically failed, resulting missing numbers in Figures 3E,F.

### GPPGPAG Penetrates to the Brain After the Oral Administration

**FIGURE 6:**
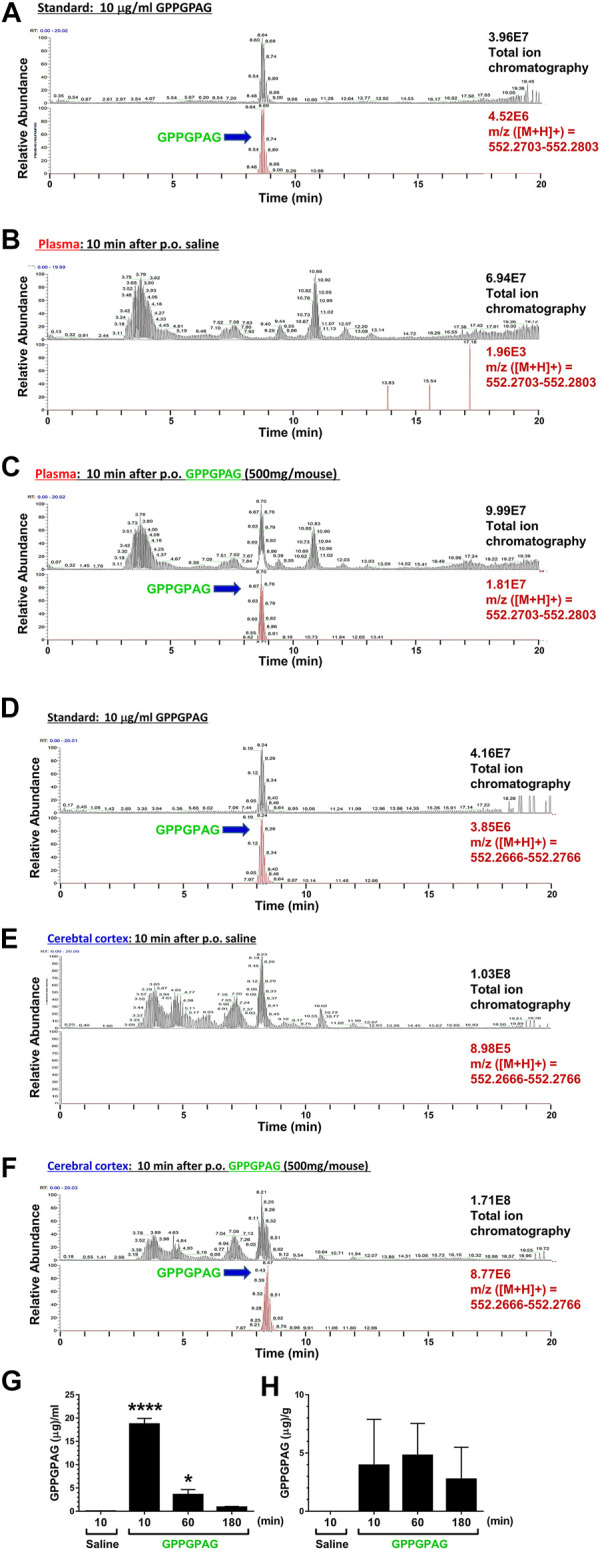
Brain penetration of GPPGPAG after oral administration in mice GPPGPAG (500 mg/mouse, p. o.) or vehicle solution (physiological saline) was administered to mice (ddY, females, 8 weeks old, *n* = 3) as a single dose. Mice were sacrificed 10, 60, or 180 min after the treatment, and plasma and cerebral cortex was extracted with methanol and subjected to LC-MS analysis. **(A)** Total ion chromatogram (upper) and extracted ion current chromatogram (lower) of standard GPPGPAG (m/z = 552.2753) at 10 μg/ml for quantification of **(B)** and **(C)** data. **(B)** Plasma sample at 10 min after administration of saline. In total ion chromatogram (upper) and extracted ion current chromatogram (lower) of m/z = 552.2753, no peaks were detected. **(C)** Plasma sample at 10 min after administration of GPPGPAG. In total ion chromatogram and extracted ion current chromatogram of m/z = 552.2753, peaks matching the retention time of GPPGPAG were detected. **(D)** Total ion chromatogram (upper) and extracted ion current chromatogram (lower) of standard GPPGPAG (m/z = 552.2716) at 10 μg/ml for quantification of (E) and (F) data. **(E)** Brain sample at 10 min after administration of saline. In total ion chromatogram (upper) and extracted ion current chromatogram (lower) of m/z = 552.2716, no peaks were detected. **(F)** Brain sample at 10 min after administration of GPPGPAG. In total ion chromatogram and extracted ion current chromatogram of m/z = 552.2716, peaks matching the retention time of GPPGPAG were detected. Quantified amount of GPPGPAG in the plasma **(G)** and cerebral cortex **(H)** are shown. *p < 0.05, ****p < 0.0001 vs, saline 10 min, one-way ANOVA with *post hoc* Bonferroni’s test, mean ± standard error, *n* = 3 mice.

To investigate a possibility of direct action of GPPGPAG in the brain, we tried to detect GPPGPAG in the plasma and cerebral cortex. Female mice (8 weeks old) were used. Using high-accuracy quasi-molecular ion ([M + H]+) and a mass error of ± 1 mmu, we detected GPPGPAG in both samples 10, 60, and 180 min after oral administration by comparing their MS-MS data and the fragmentation patterns with a reference standard (Figure 6A,D). We further profiled the chemicals that were delivered into the blood and brain in mice with HPLC-FT-MS. Usually higher than the effective dose is administered to detect the compound that is transferred to the brain, due to the small quantity of the target compound and detection limits (Durairajan et al., 2012; Tan et al., 2013; Yang et al., 2017). Therefore, we orally administered a high dose of GPPGPAG (500 mg/mouse). GPPGPAG in the plasma acutely peaked at 10 min after p. o. and reduced subsequently (Figures 6B,C,G). GPPGPAG in the cerebral cortex was detected 10 min later (Figures 6E,F,H). These results suggest that the GPPGPAG can be absorbed transiently in the systemic circulation and can penetrate to the brain after oral administration. In male mouse (9 weeks old), GPPGPAG was similarly detected in the plasma and cerebral cortex after p. o. administration (data not shown). Since biological fluids contain several peptidases, we evaluated the stability of GPPGPAG in the plasma and lysate of cerebral cortex (Supplementary Figure S3). Although an amount of GPPGPAG in saline was not declined during 180 min-incubation (Supplementary Figure S3A), GPPGPAG in the plasma was gradually reduced after 60-min incubation (Supplementary Figure S3B). By an incubation with the cerebral cortex lysate, GPPGPAG was declined at 60 min after the incubation (Supplementary Figure S3C). These results might indicate that GPPGPAG after entering the brain retained for 10 min at the longest.

### GPPGPAG Infusion to Lateral Ventricle Ameliorates Memory Impairment in AD Model Mice

To investigate the effect of direct brain delivery of GPPGPAG on the impaired object recognition memory in 5XFAD mice or wild-type mice (male and female, 24–27 weeks old), we administered GPPGPAG or the vehicle solution (ACSF) into the lateral ventricle using a micro-osmotic pump for 28 days. The concentration of GPPGPAG was maintained at approximately 10 nM in the CSF during the administration period. After 28-days of administration, an object recognition test was conducted. The interval time between the training session and a test session was set to 3 h. In the training session, all groups showed an approximately 50% preferential index (Figure 4A). In the test session, vehicle solution-infused 5XFAD mice showed around 50% of exploratory behavior, indicating a memory deficit. In contrast, vehicle solution-infused wild-type mice and GPPGPAG-infused 5XFAD mice showed significantly more frequent exploratory behavior in the presence of a novel object than that predicted by chance (50%) (Figure 4A). Repeated measures two-way ANOVA indicated significant drug × test interaction as follows.

• vehicle solution-treated wild-type vs. vehicle solution-treated 5XFAD [F (1, 7) = 9.182, P = 0.0191)
• 10 nM GPPGPAG-treated 5XFAD vs. vehicle solution-treated 5XFAD (F (1, 7) = 10.09, P = 0.0156)].

When compared with *post hoc* Bonferroni’s multiple comparisons test indicated significant improvement of object recognition memory in 10 nM GPPGPAG infused mice as well as wild-type mice (Figure 4A). The body weight was not significantly changed by GPPGPAG administration (Figure 4C). Locomotor behavior in an open field was also affected by GPPGPAG infusion (Figure 4B). In separately performed experiment with same drug administration protocol, object recognition memory test was performed with 24 h interval time between the training and test sessions using female 5XFAD mice (Supplementary Figure S2). GPPGPAG-infused 5XFAD mice showed significant improvement of object recognition memory. Although Figure 4 used mix of males and females, GPPGPAG infusion showed memory recovery at same extent in both sexes (data not shown).

### GPPGPAG Targets 14-3-3ε in Cultured Primary Neurons

To explore the mechanism responsible for GPPGPAG-induced dendrite growth and memory improvement, we used the DARTS method (Lomenick et al., 2009; Pai et al., 2015) to identify the target protein of GPPGPAG. When a compound binds to a target protein, the protein conformation is probably changed, resulting in protease sensitivity of the target protein (Lomenick et al., 2009). Primary cultured neurons were treated with a vehicle solution or GPPGPAG (10 nM) for 30 min and the cell lysate was prepared. After DARTS reaction, the protein pattern was compared on SDS-PAGE gel after silver staining. At around 28 k molecular weight, a band was thinner (facilitated proteolysis by thermolysin) after GPPGPAG treatment (Figure 5A; Supplementary Figure S4). The band was analyzed by nanoLC-MS/MS, the results indicated with high possibility that the band was 14-3-3ε (Score: 425, coverage 58%). To confirm 14-3-3ε as the target protein of GPPGPAG, we performed immunoprecipitation and dot blot using cell lysates from the peptide-treated cultured cortical neurons (Figure 5B). After GPPGPAG or vehicle solution treatment, each neuron lysate was immunoprecipitated with anti-14-3-3ε antibody. The precipitated solution was dot blotted with anti-GPPGPAG antibody and the spot intensity was quantified. GPPGPAG-treated lysate showed higher coprecipitation of 14-3-3ε and GPPGPAG, suggesting that GPPGPAG entered the neurons and bound to 14-3-3ε. The neuronal lysate was incubated with GPPGPAG or the vehicle solution, and then the DARTS reaction was performed. After gel separation on SDS-PAGE, 14-3-3 protein level was quantified by western blotting (Supplementary Figure S5). After incubation with GPPGPAG, the level of 14-3-3β/ε/ζ decreased.

14-3-3ε is an intracellular protein. If 14-3-3ε is the target molecule of GPPGPAG, GPPGPAG needs to enter the neurons. The entry of GPPGPAG inside the neurons was investigated by nanoLC-MS/MS detection. GPPGPAG entry into the neurons was detected from 10 min after treatment and gradually increased up to 360 min afterward (Supplementary Figure S6).

To investigate whether 14-3-3 mediates the GPPGPAG-induced dendrite extension, 14-3-3ε was knocked down using siRNA transfection. One day after transfection of siRNA targeting the 14-3-3ε, cultured cortical neurons were treated with Aβ1-42 and GPPGPAG simultaneously. Since a part of neurons were silenced by 14-3-3ε siRNA, we divided the 14-3-3ε siRNA-treated group as two categories, silenced cells (low level of 14-3-3ε) and other cells (unaffected cells) (Figure 5C). In the 14-3-3ε siRNA transfected group, 25.4% of cells were silenced, however, control siRNA transfection did not silence any cells. Therefore, in the 14-3-3ε siRNA-treated cells, dendritic density was separately evaluated both in the silenced cells and the other cells (Figure 5D). In control siRNA-transfected neurons, the dendritic length was significantly reduced by Aβ1-42 treatment (black column), and GPPGPAG treatment recovered the dendritic length (green column). In contrast, GPPGPAG could not enhance the dendritic length in 14-3-3ε knocked down cells (blue column), but in other cells (light blue column). These data indicate that 14-3-3ε is a target of GPPGPAG and is essential for GPPGPAG-induced dendrite growth.

## Discussion

This study shows for the first time that a heptapeptide, GPPGPAG has memory recovery effects in 5XFAD mice (Figures 3, 4). GPPGPAG treatment resulted in dendrite-specific repair (Figures 1B,C) and an increase in synaptic density (Figure 2). Whether it was orally administered or i.c.v. infused, GPPGPAG aided memory recovery in the 5XFAD mice. We also observed that orally administered GPPGPAG transferred to the brain after absorbance in the systemic circulation (Figure 6). A direct target molecule of GPPGPAG in neurons was identified as 14-3-3ε. Knockdown experiments suggested that 14-3-3ε is involved in the GPPGPAG-driven dendritic growth in the neurons (Figure 5). When administered orally, GPPGPAG increased body weights in 5XFAD mice (Figure 3C). Since food intake was not checked in this experiment, the reason why GPPGPAG affected the body weight is unknown.

Although several dipeptides such as Gly-Pro and Tyr-Pro (Tanaka et al., 2019) are known to be transported into brain, there have been no studies on GPPGPAG as a brain-transportable and a biologically active peptide. In Figure 6H, the mean value of GPPGPAG in the cerebral cortex is 2.748 μg/g of tissue at 180 min after p.o. administration of 500 mg/mouse. When 10 mg/kg GPPGPAG is applied per orally, the transferred amount of GPPGPAG in the brain is calculated as 0.598 pmol. On the other hand, the effective i.c.v dose of GPPGPAG was 10 nM (Figure 4) concentration of GPPGPAG in the pump; 1.64 μM/L, flow rate; 0.11 μL/h). Calculation from that value estimates infused amount of GPPGPAG in the ventricle for 180 min as 0.541 pmol. The brain level of GPPGPAG observed after p. o. administration is comparable to the direct infused dose of GPPGPAG. Therefore, when GPPGPAG was applied per orally, sufficient amount of GPPGPAG would reach the brain to recover the memory function. However, as shown in Supplementary Figure S3, the stability of GPPGPAG in biological fluids is not sufficient. To improve effects of GPPGPAG to more long-lasting with lower orally dosing, modifying structure for peptidase-resistant is required.

A 12-amino acid peptide (RPRTRLHTHRNR-NH_2_) crosses the blood-brain barrier (BBB) without digestion after per oral administration (Schartmann et al., 2018), although the penetration mechanism of this peptide is unknown. Identification of the BBB transporting system for GPPGPAG should be done in future.

As shown in Figure 1, GPPGPAG specifically enhanced dendritic growth, but not axonal growth. Interestingly, the active peptide was only GPPGPAG, whereas GPPGPPG or a partial sequence of GPPGPAG was not effective in dendritic formation. It implied that the sequence structure of GPPGPAG might recruit a specific target molecule in the signal pathway. By DARTS analysis, 14-3-3ε protein was demonstrated to directly bind to GPPGPAG and 14-3-3ε was involved in GPPGPAG-driven dendritic growth (Figure 5). 14-3-3ε forms a homodimer or a heterodimer with other subtypes of 14-3-3. The family of 14-3-3 proteins is abundant in the brain and mediate a wide variety of cellular events such as cell cycle regulation, cell proliferation, and membrane trafficking (Darling et al., 2005). Dendrite formation activity of 14-3-3ε has been reported previously, heterodimer of 14-3-3ε/ζ stabilizes δ-catenin, resulting in enhanced dendrogenesis (He et al., 2012). δ-catenin regulates the maintenance of dendrites and dendritic spines in the mature cerebral cortex (Matter et al., 2009). Although it is unknown if binding of GPPGPAG to 14-3-3ε affects δ-catenin function, we focus on the δ-catenin-mediated dendrogenesis as the GPPGPAG pathway. Although we need to verify 14-3-3ε-mediated dendrite formation *in vivo* in the next step, considering that 14-3-3ε knockout mouse revealed impairment of object memory (Wachi et al., 2017), regulation of 14-3-3ε function and/or expression is possibly related to memory function. Other subtypes of 14-3-3, such as β, γ, and ζ were reported as stimulators of axonal growth (Kaplan et al., 2017). In the case, small molecule fusicoccin-A stabilizes interaction between 14-3-3 and stress response regulator GCN1, leading axonal growth. In contrast, axonal growth by 14-3-3ε has not been addressed yet. If 14-3-3ε would regulate specifically dendrites, GPPGPAG might stabilize the interaction of 14-3-3ε and δ-catenin. 14-3-3 proteins bind to numerous partner proteins. As peptide ligands of 14-3-3ε, CBP501, a chemically modified twelve unnatural D-amino acids binds to 14-3-3ε, although how the binding affects 14-3-3ε function is unknown (Matsumoto et al., 2011).

Aβ-induced disruption of neuronal networks is a well-recognized phenomenon. As abnormal dendrite morphology by Aβ plaques, spine loss, shaft atrophy and bending are frequently observed in the AD model mice (Dickson and Vickers, 2001). In early phase APP/PS1 mice, recovery of spine density by LTP stimulation restored the memory loss (Grutzendler et al., 2007). This report suggests that dendritic density may be closely associated with memory formation. The morphological restoration of dendrites might be partially possible in an early phase of AD, but it is likely hard in an later phase. In contrast, the present study showed that dendritic repair by GPPGPAG treatment occurs even during the progressive phase in 5XFAD mice (Jiang et al., 2015). GPPGPAG administration improved memory function without reducing Aβ plaques (Figures 3E,F), supposing that the recovery effect on cognitive function is mediated by neuronal network reconstruction. We will further investigate GPPGPAG effects on several cognitive properties using other types of memory tests in future.

In conclusion, our results indicated that GPPGPAG could promote recovery from memory dysfunction in AD model mice, and this effect may be mediated by dendritic repair. This novel finding may lead to development of new anti-AD medicines which help in reconstructing neuronal circuits by restoring dendritic atrophy.

## Data Availability

The original contributions presented in the study are included in the article/Supplementary Material, further inquiries can be directed to the corresponding author.

## References

[B1] Bertoni-Freddari C. Fattoretti P. Solazzi M. Giorgetti B. Di Stefano G. Casoli T. (2003). Neuronal Death versus Synaptic Pathology in Alzheimer's Disease. Ann. N.Y. Acad. Sci. 1010, 635–638. 10.1196/annals.1299.116 15033803

[B2] Darling D. L. Yingling J. Wynshaw‐Boris A. (2005). Role of 14-3-3 Proteins in Eukaryotic Signaling and Development. Curr. Top. Dev. Biol. 68, 281–315. 10.1016/S0070-2153(05)68010-6 16125003

[B3] Dickson T. C. Vickers J. C. (2001). The Morphological Phenotype of β-amyloid Plaques and Associated Neuritic Changes in Alzheimer's Disease. Neuroscience 105, 99–107. 10.1016/s0306-4522(01)00169-5 11483304

[B4] Durairajan S. S. K. Liu L.-F. Lu J.-H. Chen L.-L. Yuan Q. Chung S. K. (2012). Berberine Ameliorates β-amyloid Pathology, Gliosis, and Cognitive Impairment in an Alzheimer’s Disease Transgenic Mouse Model. Neurobiol. Aging 33, 2903–2919. 10.1016/j.neurobiolaging.2012.02.016 22459600

[B5] Eriksen J. L. Janus C. G. (2007). Plaques, Tangles, and Memory Loss in Mouse Models of Neurodegeneration. Behav. Genet. 37, 79–100. 10.1007/s10519-006-9118-z 17072762

[B6] Grutzendler J. Helmin K. Tsai J. Gan W.-B. (2007). Various Dendritic Abnormalities Are Associated with Fibrillar Amyloid Deposits in Alzheimer's Disease. Ann. N Y Acad. Sci. 1097, 30–39. 10.1196/annals.1379.003 17413007

[B7] He Y. Han J. R. Chang O. Oh M. James S. E. Lu Q. (2012). 14-3-3ɛ/ζ Affects the Stability of δ-catenin and Regulates δ-catenin-induced Dendrogenesis. FEBS Open Bio 3, 16–21. 10.1016/j.fob.2012.11.006 PMC3668525 23772369

[B8] Jiang X. Chai G.-S. Wang Z.-H. Hu Y. Li X.-G. Ma Z.-W. (2015). Spatial Training Preserves Associative Memory Capacity with Augmentation of Dendrite Ramification and Spine Generation in Tg2576 Mice. Sci. Rep. 5, 9488. 10.1038/srep09488 25820815 PMC4377552

[B9] Joyashiki E. Matsuya Y. Tohda C. (2011). Sominone Improves Memory Impairments and Increases Axonal Density in Alzheimer's Disease Model Mice, 5XFAD. Int. J. Neurosci. 121, 181–190. 10.3109/00207454.2010.541571 21329473

[B10] Kaplan A. Morquette B. Kroner A. Leong S. Madwar C. Sanz R. (2017). Small-molecule Stabilization of 14-3-3 Protein-Protein Interactions Stimulates Axon Regeneration. Neuron 93 (5), 1082–1093. 10.1016/j.neuron.2017.02.018 28279353

[B11] Kogure C. Tohda C. (2017). Human Placenta Extract Ameliorates Memory Dysfunction and Dendritic Atrophy in a 5XFAD Mouse Model of A Lzheimer's Disease. Traditional Kampo Med. 4, 94–104. 10.1002/tkm2.1075

[B12] Lomenick B. Hao R. Jonai N. Chin R. M. Aghajan M. Warburton S. (2009). Target Identification Using Drug Affinity Responsive Target Stability (DARTS). Proc. Natl. Acad. Sci. 106, 21984–21989. 10.1073/pnas.0910040106 19995983 PMC2789755

[B13] Matsumoto Y. Shindo Y. Takakusagi Y. Takakusagi K. Tsukuda S. Kusayanagi T. (2011). Screening of a Library of T7 Phage-Displayed Peptides Identifies alphaC Helix in 14-3-3 Protein as a CBP501-Binding Site. Bioorg. Med. Chem. 19 (23), 7049–7056. 10.1016/j.bmc.2011.10.004 22032894

[B14] Matter C. Pribadi M. Liu X. Trachtenberg J. T. (2009). δ-Catenin Is Required for the Maintenance of Neural Structure and Function in Mature Cortex *In Vivo* . Neuron 64, 320–327. 10.1016/j.neuron.2009.09.026 19914181 PMC2840037

[B15] Oakley H. Cole S. L. Logan S. Maus E. Shao P. Craft J. (2006). Intraneuronal Beta-Amyloid Aggregates, Neurodegeneration, and Neuron Loss in Transgenic Mice with Five Familial Alzheimer's Disease Mutations: Potential Factors in Amyloid Plaque Formation. J. Neurosci. 26, 10129–10140. 10.1523/JNEUROSCI.1202-06.2006 17021169 PMC6674618

[B16] Pai M. Y. Lomenick B. Hwang H. Schiestl R. McBride W. Loo J. A. (2015). Drug Affinity Responsive Target Stability (DARTS) for Small-Molecule Target Identification. Methods Mol. Biol. 1263, 287–298. 10.1007/978-1-4939-2269-7_22 25618353 PMC4442491

[B17] Schartmann E. Schemmert S. Ziehm T. Leithold L. H. E. Jiang N. Tusche M. (2018). Comparison of Blood-Brain Barrier Penetration Efficiencies between Linear and Cyclic All-D-Enantiomeric Peptides Developed for the Treatment of Alzheimer's Disease. Eur. J. Pharm. Sci. 114, 93–102. 10.1016/j.ejps.2017.12.005 29225107

[B18] Tan X.-S. Ma J.-Y. Feng R. Ma C. Chen W.-J. Sun Y.-P. (2013). Tissue Distribution of Berberine and its Metabolites after Oral Administration in Rats. PLoS ONE 8, e77969. 10.1371/journal.pone.0077969 24205048 PMC3815028

[B19] Tanaka M. Dohgu S. Dohgu S. Komabayashi G. Kiyohara H. Takata F. (2019). Brain-transportable Dipeptides across the Blood-Brain Barrier in Mice. Sci. Rep. 9, 5769. 10.1038/s41598-019-42099-9 30962462 PMC6453885

[B20] Tohda C. Urano T. Umezaki M. Nemere I. Kuboyama T. (2012). Diosgenin Is an Exogenous Activator of 1,25D3-MARRS/Pdia3/ERp57 and Improves Alzheimer's Disease Pathologies in 5XFAD Mice. Sci. Rep. 2, 535. 10.1038/srep00535 22837815 PMC3405293

[B21] Wachi T. Cornell B. Toyo-Oka K. (2017). Complete Ablation of the 14-3-3epsilon Protein Results in Multiple Defects in Neuropsychiatric Behaviors. Behav. Brain Res. 319, 31–36. 10.1016/j.bbr.2016.11.016 27845227 PMC5183496

[B22] Warburton E. C. Brown M. W. (2015). Neural Circuitry for Rat Recognition Memory. Behav. Brain Res. 285, 131–139. 10.1016/j.bbr.2014.09.050 25315129 PMC4383363

[B23] Yang Z. Kuboyama T. Tohda C. (2017). A Systematic Strategy for Discovering a Therapeutic Drug for Alzheimer's Disease and its Target Molecule. Front. Pharmacol. 8, 340. 10.3389/fphar.2017.00340 28674493 PMC5474478

