# Supplementary material for: A Novel Heptapeptide, GPPGPAG Transfers to the Brain, and Ameliorates Memory Dysfunction and Dendritic Atrophy in Alzheimer’s Disease Model Mice
Source: Front Pharmacol. 2021 May 14;12:680652. doi: 10.3389/fphar.2021.680652 (PMC8160438; doi:10.3389/fphar.2021.680652)
Supplement: Supplementary file 1 [file DataSheet1.pdf]

Supplementary Information

A novel heptapeptide, GPPGPAG transfers to the brain, and ameliorates memory dysfunction and dendritic atrophy in Alzheimer's disease model mice

Chihiro Tohda[1]*, Chisato Kogure[1], Kaori Nomoto[1,2], Andreia de Toledo[2], Ximeng Yang[1] & Eiichi Hirano[2]

1 Section of Neuromedical Science, Division of Bioscience, Institute of Natural Medicine, University of Toyama, Toyama, Japan
2 Research Institute Japan Bio Products Co., Ltd. Kurume, Japan.

Chihiro Tohda, Chisato Kogure and Kaori Nomoto contributed equally.

*Correspondence: Chihiro Tohda, Ph.D.
Section of Neuromedical Science, Division of Bioscience, Institute of Natural Medicine, University of Toyama, 2630 Sugitani, Toyama 930-0194, Japan
E-mail: chihiro@inm.u-toyama.ac.jp
Phone: +81-76-434-7646

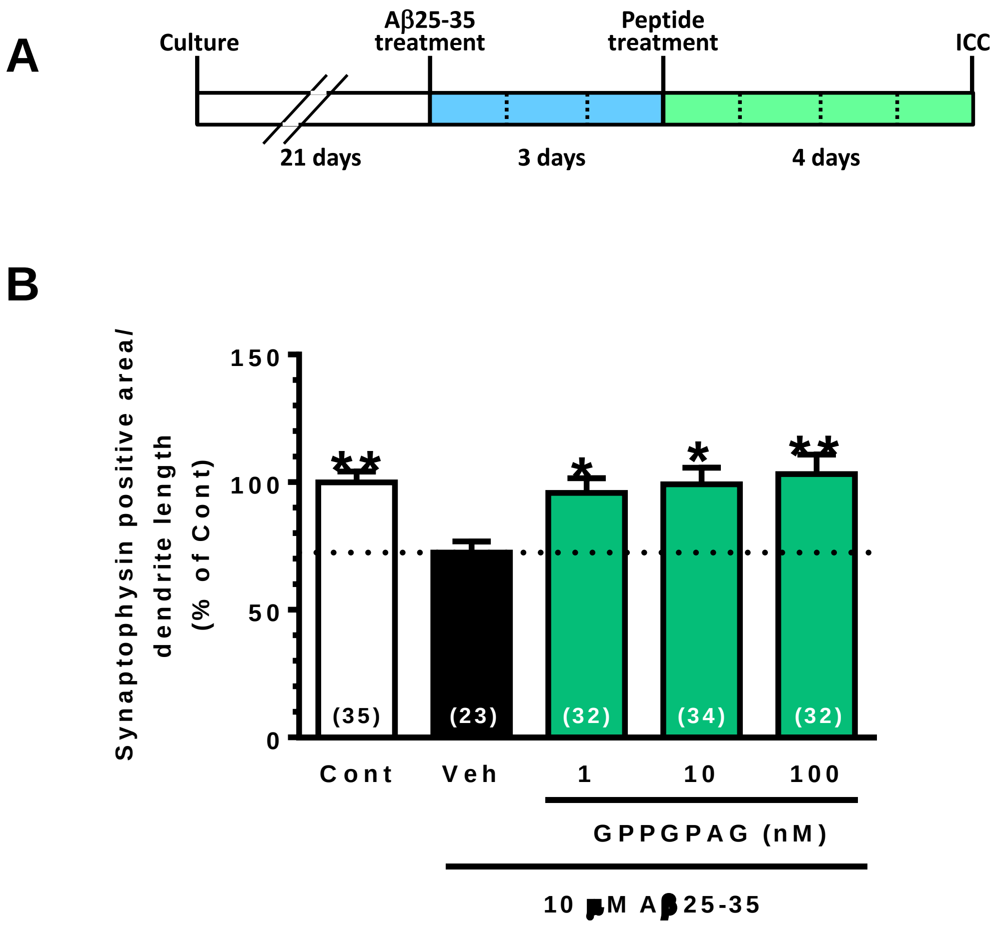

Supplementary Figure 1
Effect of GPPGPAG on Aβ-induced pre-synaptic loss.
(A) Cortical neurons were cultured for 21 days and then treated with or without aggregated Aβ(25-35) (10 μM). Three days after the administration of Aβ25-35, the cells were treated with GPPGPAG or a vehicle solution (distilled) water. After an additional 4-days of treatment, the cells were fixed and double-immunostained for MAP2 and synaptophysin. (B) The synaptophysin-positive puncta area was quantified for each dendrite. *P < 0.05, **P < 0.01 vs Aβ25-35/Veh, one-way ANOVA with *post hoc* Bonferroni's test, n = 23 - 35.

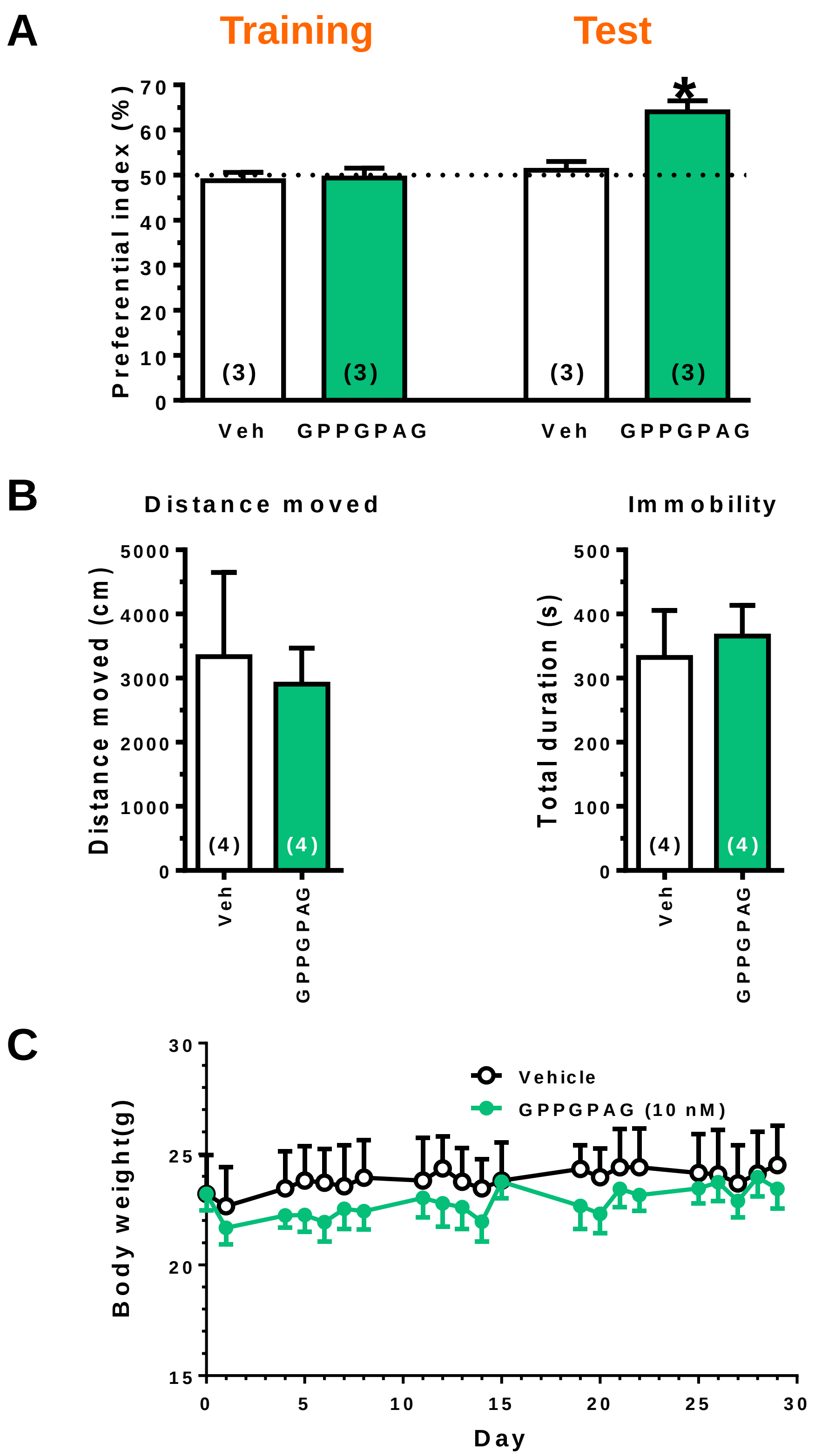

Supplementary Figure 2

Effect of i.c.v. infusion of GPPGPAG on object recognition memory deficits in 5XFAD mice (A) GPPGPAG or vehicle solution (artificial cerebrospinal fluid; ACSF) was continuously administered to the lateral ventricle for 28 days in mice (females, 5 - 7 months old). The dose of GPPGPAG was set as 10 nM in ACSF. An object recognition test was carried out with a 24 h interval time. The preferential indices of the training and test sessions are shown. *P < 0.05 vs. the same group in the training session, paired t-test; n = 3 mice). (B) Locomotion was evaluated by open field test. Distance moved and duration with immobility were shown. (C) Body weight of mice during whole experimental period are shown. Repeated measures two-way ANOVA revealed no significant difference among four groups.

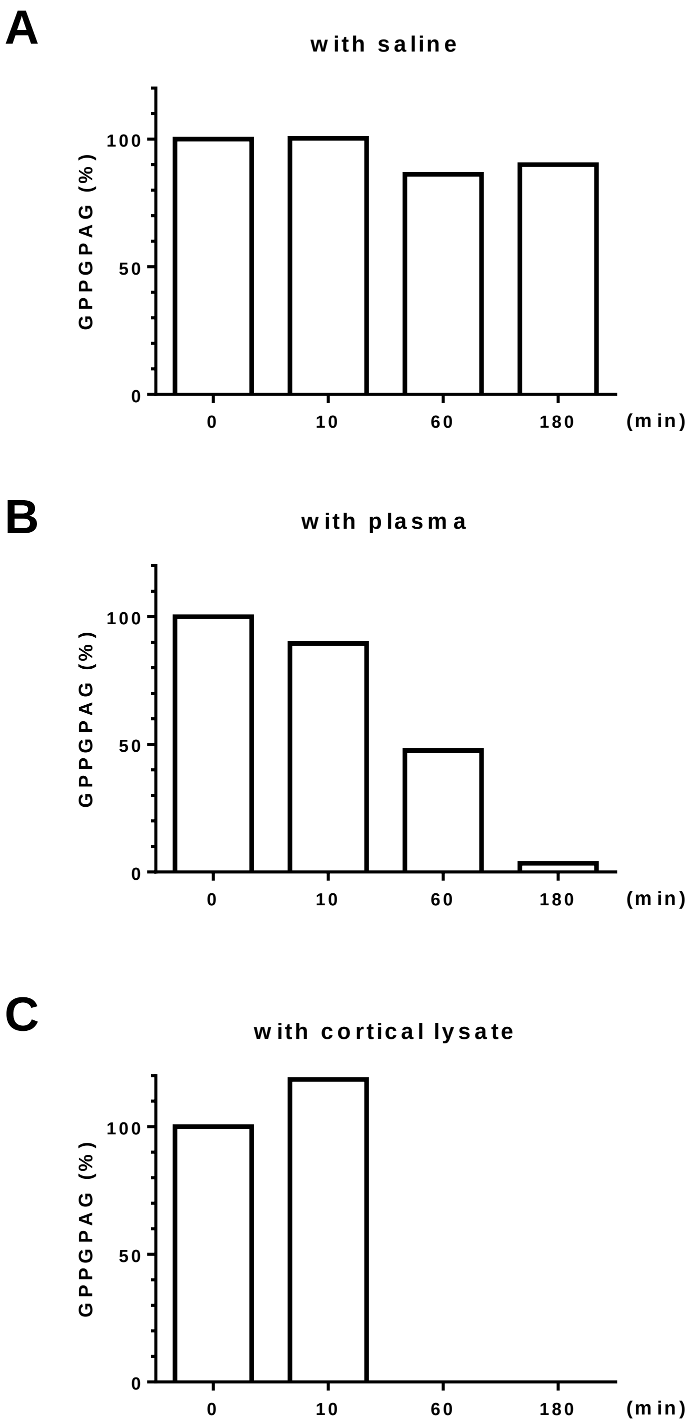

Supplementary Figure 3
Stability of GPPGPAG in biological fluids.
(A) GPPGPAG was mixed with saline at 5 µg/ml concentration. (B) GPPGPAG was mixed with mouse plasma at 5 µg/ml concentration. (C) GPPGPAG was mixed with fresh lysate of mouse cerebral cortex at 6.5 µg/g of cortex. After incubation at 37° C for 0, 10, 60 and 180 min, samples were dried up and served to LC-MS. Areas of MS peaks of GPPGPAG were quantified. n = 1.

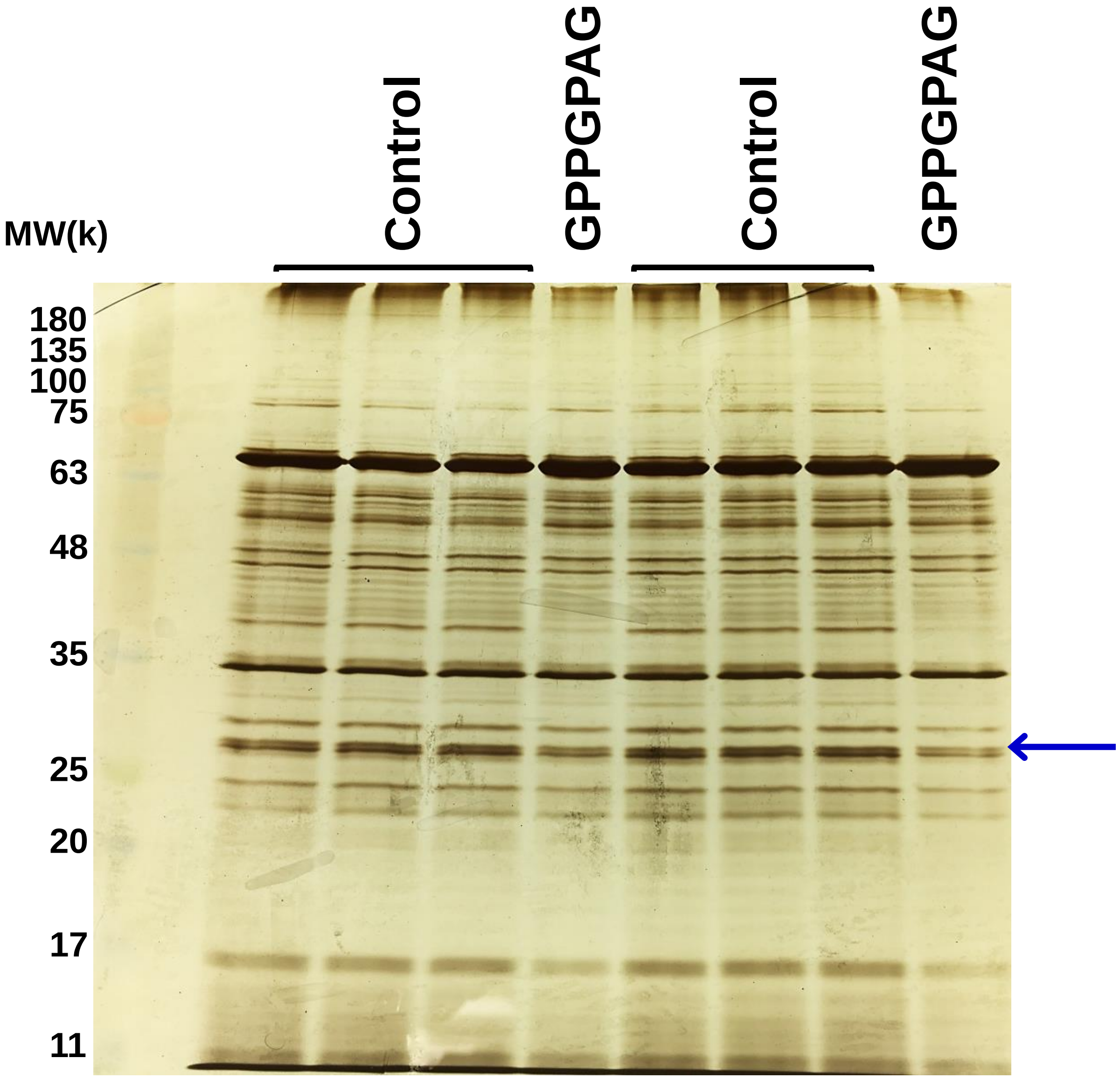

Supplementary Figure 4
DARTS analysis of mouse cortical neurons was performed using cultured cortical neurons. After treatment with vehicle solution (water) or 10 nM GPPGPAG for 30 min at 37℃, cell lysate was prepared, and thermolysin reaction was performed. At 28 k, the band level in GPPGPAG-treated neuronal lysate was thinner compared with the vehicle solution-treated group.

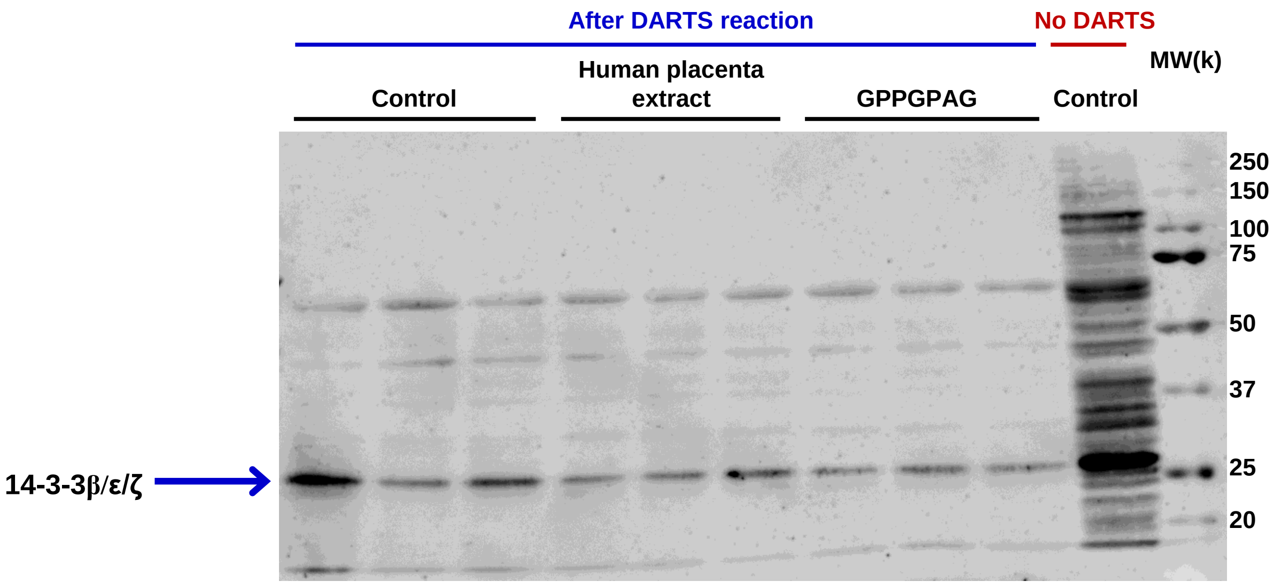

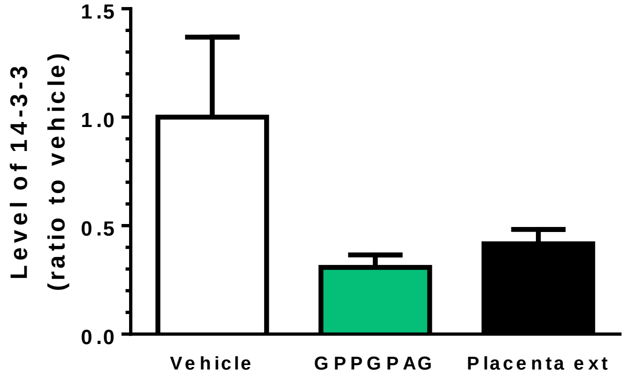

Supplementary Figure 5

DARTS analysis and then western blotting for 14-3-3 protein were performed using mouse cortical neurons. After treatment neuronal lysate with vehicle solution (water), 10 nM GPPGPAG or positive control 10 μg/ml human placenta extract for 30 min at room temperature, thermolysin reaction was performed. After SDS-PAGE, gel was transferred to a nitrocellurose membrane, western blotting for 14-3-3β/ε/ζ was performed. Without DARTS reaction, undigested protein bands were loaded on the lane (No DARTS). The band level in GPPGPAG-treated neuronal lysate was thinner compared with the vehicle solution-treated group.

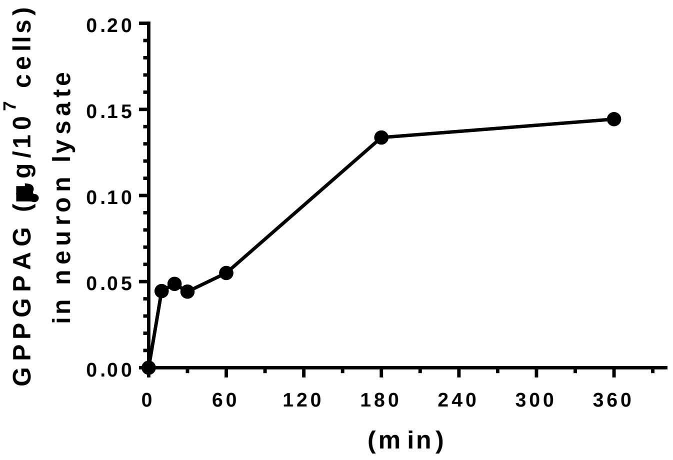

Supplementary Figure 6
Intracellular amount of GPPGPAG after addition to cultured cortical neurons. Mouse cortical neurons were seeded at 1.0 x 10^7 cells/10-cm dish. After 3 – 4 days culture, 1 mg/dish GPPGPAG was added. Incubation time was 0, 10,20, 30, 60, 180 and 360 min. After complete washing by PBS, cell lysate was prepared by M-PER solution. The lysate was ultrasonicated with methanol for 60 sec, and centrifuged (12000g) for 10 min at 4°C. Supernatant was dried up and served for LC-MS analysis.
